# Potential effects of sweet almond oil in the treatment of Alzheimer’s disease targeting BDNF and TrkB: An *In Silico* Molecular docking and ADMET Evaluation Study

**DOI:** 10.1002/alz.088768

**Published:** 2025-01-09

**Authors:** Rahmat Hassan Salis, Uduak Emmanuel Umana, Adamu Abubakar Sadeeq

**Affiliations:** ^1^ Ahmadu Bello University, Zaria, Kaduna Nigeria

## Abstract

**Background:**

Alzheimer’s disease (AD) is the most prevalent cause of dementia accounting for an estimated 60% to 80% of cases. Despite advances in the research field, developing truly effective therapies for AD symptoms remains a major challenge. Sweet almond contain nutrients that have the potential of combating age‐related brain dysfunction, by improving learning, memory and neurocognitive performance. Neurotrophins are molecules that support neuroplasticity and are capable of promoting neuronal survival, controlling cell proliferation and neuronal differentiation. Brain‐derived neurotrophic factor (BDNF) is the most abundant neurotrophic factor that signals through the tropomycin receptor kinase B (TrkB). It plays a critical role in learning and memory by promoting survival of neurons and modulating synaptic connectivity. Loss of BDNF/TrkB signaling has been reported to play a role in AD, suggesting a pathologic involvement.

**Method:**

42 compounds in sweet almond oil were identified using GC‐MS analysis. The 2D structures of all the compounds were retrieved from PubChem database and the 3D structures of BDNF (PDB: 1B8M) and TrkB (PDB: 1WWB) were downloaded from RCSB database. BDNF and TrkB were prepared using PyMOL software and ligand preparation/docking were carried out using PyRx software. ADMET evaluation of the compounds was performed using ADMETlab 2.0 server.

**Result:**

12 compounds were found to have a higher binding affinity than Memantine, a drug that was reported to upregulate BDNF/TrkB expression in the brain. The docking results revealed that CID‐2116, CID‐6421311, CID‐5357430, CID‐541960, CID‐543355, CID‐37153, CID‐67513, CID‐543450, CID‐31039, and CID‐35698 have binding affinity of ‐11.2 kcal/mol, ‐7.4 kcal/mol, ‐6.8 kcal/mol, ‐6.6 kcal/mol, ‐6.1 kcal/mol, ‐6.0 kcal/mol, ‐6.0 kcal/mol, ‐5.8 kcal/mol, ‐5.7 kcal/mol, ‐5.7 kcal/mol respectively against BDNF that exceeded Memantine (‐5.6 kcal/mol). The result also revealed that CID‐2116, CID‐141010, CID‐31039, and CID‐549641 have binding affinity of ‐9.3 kcal/mol, ‐6.6 kcal/mol, ‐6.1 kcal/mol, and ‐6.0 kcal/mol respectively against TrkB receptor that exceeded Memantine (‐5.7 kcal/mol). All of these compound showed positive BBB penetration, 0 alert on acute toxicity rule and obeying Lipinski’s rule of five parameters.

**Conclusion:**

The results of this study revealed that some compounds of sweet almond oil have potential therapeutic activity against BDNF/TrkB expression with low systematic toxicity.

